# Contribution of Electrolyte Decomposition Products and the Effect of Temperature on the Dissolution of Transition Metals from Cathode Materials

**DOI:** 10.1021/acsomega.3c03173

**Published:** 2023-08-25

**Authors:** Janik Lüchtefeld, Ming-Yu Lee, Hendrik Hemmelmann, Susanne Wachs, Christopher Behling, Karl J. J. Mayrhofer, Matthias T. Elm, Balázs B. Berkes

**Affiliations:** † Non-Aqueous Electrochemistry, Electrocatalysis Department, Helmholtz Institute Erlangen-Nürnberg for Renewable Energy (IEK-11), Forschungszentrum Jülich, Cauerstr. 1, 91058 Erlangen, Germany; ‡ Department of Chemical and Biological Engineering, Friedrich-Alexander-Universität Erlangen-Nürnberg, Cauerstr. 1, 91058 Erlangen, Germany; § Center for Materials Research, Justus-Liebig-University Gießen, Heinrich-Buff-Ring 16, 35392 Gießen, Germany

## Abstract

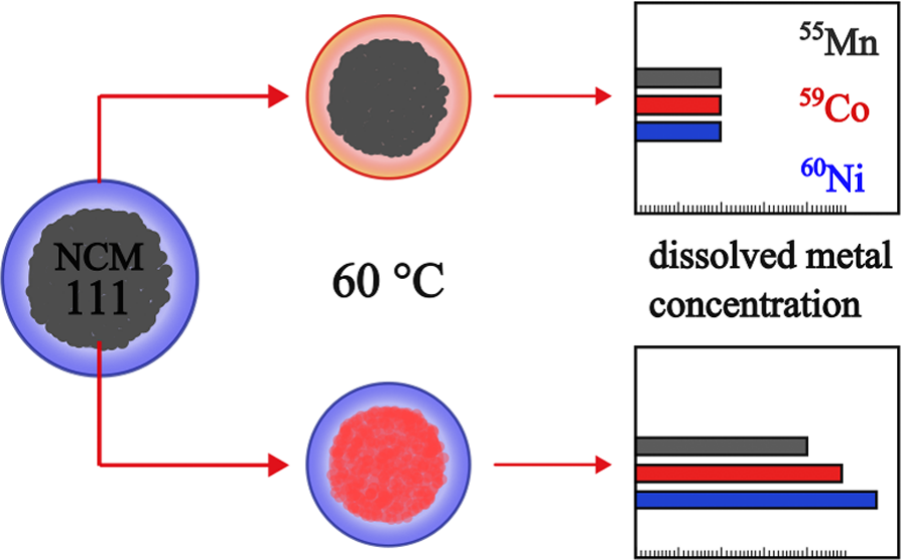

A fundamental understanding of aging processes in lithium-ion batteries (LIBs) is imperative in the development of future battery architectures for widespread electrification. Herein, dissolution of transition metals from cathode active materials of LIBs is among the most important degradation processes. Research has demonstrated that elevated operating temperatures accelerate battery degradation. However, the exact mechanism of transition-metal dissolution at elevated temperatures has still to be clarified. Current literature suggests that the reaction rate of dissolution increases with increasing temperature; moreover, the decomposition of electrolytes results in products that also accelerate dissolution processes. Most studies focus on ex situ analyses of thermally treated full cells. This approach is not appropriate to get detailed insights and to distinguish between different contributions. In this work, with the help of real-time dissolution analysis using an electroanalytical flow cell (EFC) coupled to an inductively coupled plasma mass spectrometer (ICP-MS), we present novel details of the temperature effects on in situ dissolution at the cathode electrolyte interface. With fresh electrolytes, we find increased Mn dissolution even at open-circuit conditions as well as with constant voltage polarization when the electrode sample is heated at constant temperatures between 50 and 80 °C. The release of transition metals also responds in a nuanced manner when applying temperature transients. Utilizing electrolytes preheated at 60 and 100 °C, we demonstrate that decomposition products in the bulk electrolyte have no influence on transition-metal (TM) dissolution when constantly flushing the cell with the thermally aged electrolyte samples. Only when keeping the cathode temperature at 60 °C, the dissolution increases by a factor of 2–3. Our findings highlight the interplay between the cathode and electrolyte and provide new insights into the dissolution mechanism of cathode materials.

## Introduction

The growing implementation of lithium-ion batteries in many, but especially in large-scale consumer applications, namely, electric vehicles (EV), has pushed conventional LIB cell designs and operating parameters to their limits. Demands for increased driving ranges and faster charge rates necessitate measures such as extended utilization of lithium inventory, high operating voltages, and large cycling rates—all of which are known to reduce battery lifetime by exacerbating electrochemical degradation processes.^1−4^ In order to develop suitable mitigation strategies, it is therefore crucial to gather a rigorous conception of battery aging mechanisms. An important parasitic side reaction is the dissolution of transition metals from the cathode active material (CAM). Changes in the crystal symmetry upon insertion or removal of Li ions from the host structure during cycling, known as electromechanical grinding, eventually cause particle cracking and oxygen release and facilitate the attack of leaching agents from the electrolyte.^5^ Dissolved metals subsequently deposit on the anode, trapping Li ions and thickening a resistive solid electrolyte interface (SEI). Consequently, the cell impedance increases, metallic lithium dendrites, short circuiting may occur, and significant capacity fade sets in much faster.^6^

Due to thermodynamic instability issues of battery components, like that of the liquid electrolyte, many degradation processes are accelerated at even slightly elevated temperatures, i.e., 60 °C or above. Their detrimental impact on battery performance has been demonstrated, among others, for electrolyte stability, capacity retention, cell impedance, and for CAM dissolution processes as well.^7−9^ In particular, Mn-based spinel cathodes are prone to increased dissolution upon thermal treatment, but enhanced dissolution of Co and Ni has been reported likewise.^10−13^ Suppressing high temperature degradation is of vital importance for battery applications subjected to warm ambient conditions, e.g., during summer, or in confined environments with regard to self-heating (thermal propagation of battery packs in particular). Sufficient cooling is necessary to ensure battery performance and lifetime, as well as to prevent safety issues such as swelling or ignition.^14^

In the context of elucidating dissolution mechanisms from cathode materials, the aforementioned studies exclusively provide cumulative dissolution data gathered by ex situ analyses. Although this kind of information is important in providing information on real applications, fundamental insight into transient processes or separating the contribution of single components is inherently impossible. For instance, the dissolution behavior of transition metals (TM) might be affected by thermal decomposition products in the electrolyte, which are known to evolve, as shown by comprehensive electrolyte analyses.^7^ Determination of TM concentration levels in the electrolyte of cycled cells, however, does not allow the evaluation of the impact of these compositional changes conclusively. In this study, we tackle the issue by employing our electroanalytical flow cell (EFC), which we have presented in previous publications.^15−17^ As a model electrode system, we chose LiNi_0.33_Co_0.33_Mn_0.33_O_2_ (NCM111) thin-film cathodes, as employed in our previous publication.^18,19^ The cathode working electrode is the only component directly heated in our setup as the entire cell volume of liquid is constantly replaced by electrolyte at ambient temperature (25 °C) in less than 2 s. We find that already a slight increase of the cathode temperature to 50 °C facilitates TM dissolution under moderate voltages of 4.0 vs Li^+^/Li, which drastically increases for even higher temperatures. Analysis of electrolytes preheated at 60 °C for 18 h reveals no significant changes in the electrolyte composition; however, some literature reports suggest that even low quantities of decomposition products in LiPF_6_ electrolytes, which might not be recognized with GC-MS in the bulk electrolyte, can have a significant impact on surface reactions with the cathode.^20^ It has also been demonstrated that the release of surface oxygen from the cathode catalyzes oxidation reactions of the electrolyte^21^ and that the degree of decomposition is exacerbated by elevated temperatures.^22^ Formation of decomposition products, e.g., acidic fluorine species, may then enhance dissolution of transition metals from the cathode.^23^ Considering that laminar flow regimes in the cell will create immobile electrolyte layers in direct contact with the cathode surface,^24^ complete separation of heating effects on the cathode and electrolyte and corresponding reactions are only possible for the bulk structures. Nonetheless, decreasing the impact of bulk properties enables us to focus on the interfacial interplay between the cathode surface and the surface electrolyte, i.e., operando transition-metal dissolution, and the corresponding impact of temperature with unprecedented sensitivity.

## Results and Discussion

**Figure 1:**
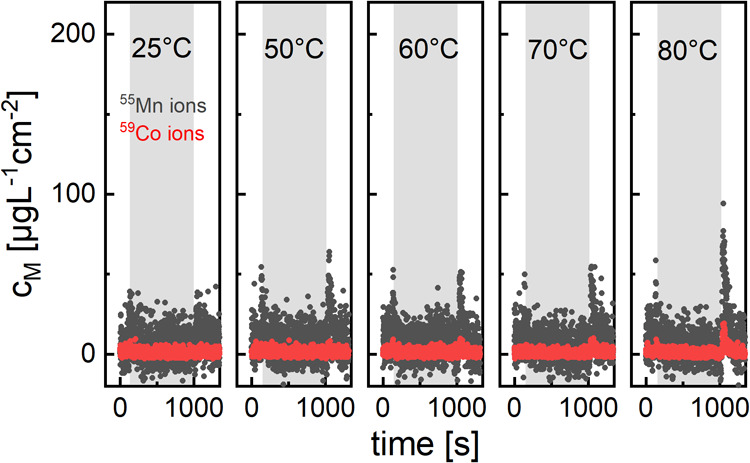
Dissolution profiles of Mn, Co, and Ni ions from a NCM111 thin-film cathode in a EFC with stagnant electrolyte at open-circuit conditions. The spots were conditioned for 15 minutes at the indicated temperature (gray background in the diagrams). Then, the electrolyte was directed through the cell again and pumped to the ICP-MS (white background of the diagrams). Ni data were omitted due to high fluctuations of the dissolution signal.

We start the examination of heating effects on TM dissolution by measuring thermal dissolution from the thin-film cathode without applied potential or a current. In EFC configuration, the temperature of the translation stage was set to 25, 50, 60, 70, and 80 °C (same sample spot), respectively, and the dissolution was monitored with ICP-MS. Once the transition-metal analytes had reached their respective baseline values at a constant temperature, the stopcock valve was switched to bypass configuration. After 15 min, the valve was switched back, and the entire EFC channel volume (ca. 40 μL) was conveyed to the ICP-MS to analyze the dissolved transition-metal concentration in the electrolyte. To mitigate additional dissolution by the hot plate, the temperature was directly lowered to 25 °C in the Peltier control software after reopening the valve to the EFC. The corresponding dissolution data are presented in Figure 1.

The gray area indicates the time frame in which the electrolyte was stagnant in the EFC, enabling accumulation of dissolved species. Switching the flow from EFC to the bypass tubing generates phantom artifacts in the dissolution profile, most notably for Ni (hence the omission in Figure 1) because the pressure loss along the bypass is lower compared to the EFC pathway (Figure S2). This disturbance proceeds to the ultimate mixing tee in which internal standard and diluted electrolyte are combined, so that more electrolyte is being introduced to the ICP-MS temporarily. Likewise, when switching back to the EFC, the analyte signal is bound to drop slightly, due to the higher flow resistance. Mn does react to the initial switch to bypass configuration, as well. Remarkably, after switching back to the EFC, a faint dissolution peak emerges even at 25 °C. The presence of Mn peaks becomes more evident after increasing the temperature to 50 °C and above, whereas Co dissolution is less intense below 80 °C. Ni dissolution, due to its higher baseline concentration and fluctuations of the signal, cannot be determined. By comparing the analyte signals with the internal standard signal, we can confirm that the peaks of Mn and Co are indeed due to dissolution (Figure S2).

**Figure 2:**
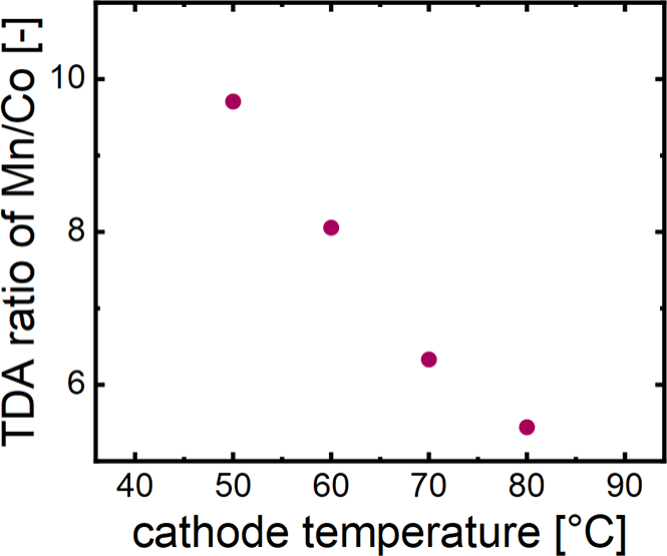
Ratio of total dissolved amounts of Mn and Co ions after 15 min of heating in bypass configuration at indicated temperatures. The dissolution levels are below the limit of quantification. Nevertheless, a clear trend in TDA increase is recognizable.

The total dissolved amount (TDA) of metal may be determined by integration of the dissolution peak and multiplication with the electrolyte flow rate (150 μL min^–1^). It should be noted that the overall metal concentration is soundly below the limit of quantification. However, we can still indicate that the TDA of Mn is approximately 10 times the value of Co at a heating plate (HP) temperature of 50 °C. In addition, the Co ion dissolution rate increases much faster with increasing temperature, i.e., the TDA of Mn is only 5 times larger than for Co at 80 °C (ca. 0.5 vs 0.1 ng), as presented in Figure 2.

**Figure 3:**
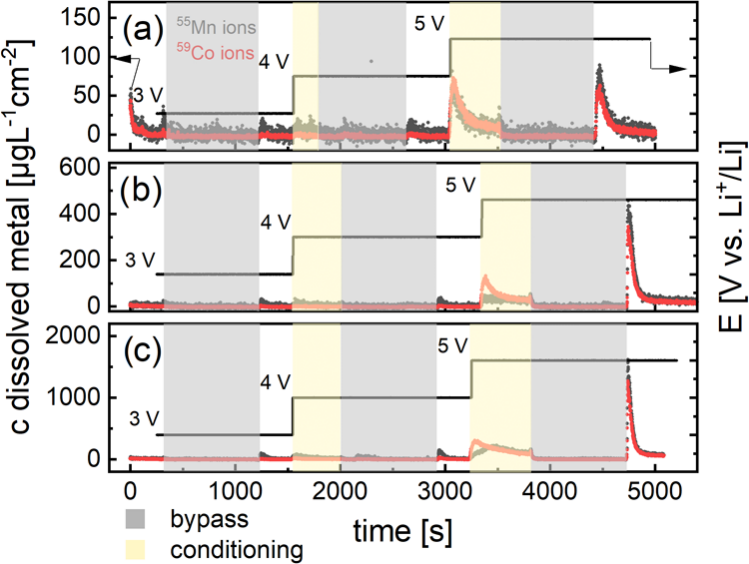
Dissolution profiles of Mn and Co at (a) 25 °C, (b) 50 °C, and (c) 80 °C in a EFC with stagnant electrolyte at 3.0, 4.0, and 5.0 V vs Li^+^/Li. At the beginning of the constant potential step, the EFC was flushed with electrolyte until constant dissolution values were achieved. Then, the cell was conditioned for 15 minutes with active bypass (gray background), before the electrolyte was directed through the cell again (white background). After reaching constant dissolution again, the potential was stepped up to the subsequent value (beige background), hence the repetition of the protocol. Note that the *y*-axis scaling increases by an order of magnitude from (a) 150 to (c) 2000 μg L^–1^ cm^–2^.

Based on the results of the open-circuit experiments, we repeated the measurements with the bypass setup, but we applied a constant potential to the cathode for a 15 min accumulation period. The thin-film cathode was kept at 25, 50, and 80 °C, respectively, and the potential was consecutively increased stepwise to 3.0, 4.0, and 5.0 V vs Li^+^/Li. As soon as baseline dissolution was achieved at each potential, the electrolyte flow was switched to the bypass configuration. After 15 min, the stopcock was switched to the EFC again, and the cell volume was transferred to the ICP-MS. The individual dissolution data are presented in Figure 3. Please note that the Ni profiles have been omitted due to the overall low response of the ICP-MS to this metal below 5.0 V vs Li^+^/Li. The corresponding dissolution graphs are presented in the SI (Figures S3–S5).

**Figure 4:**
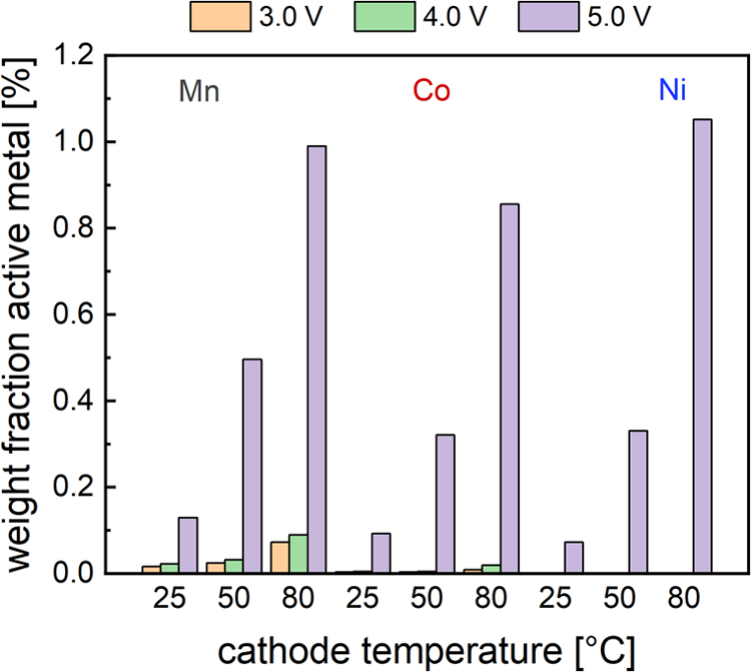
Dissolved percentage of total active metal of Mn, Co, and Ni ions after 15 min of polarization at indicated potentials and temperatures in an EFC with stagnant electrolyte flow.

A high voltage polarization of 5.0 V vs Li^+^/Li significantly increases the TM dissolution for all tested temperatures. It is also apparent that Mn dissolution is more pronounced than Co dissolution throughout all heating and polarization conditions (peak of the gray curve directly after a gray background in Figure 3), in agreement with the previous open-circuit voltage (OCV) experiments shown in Figure 1. The dissolved Co concentration for a voltage polarization of 3.0 and 4.0 V vs Li^+^/Li is just at the limit of detection at 25 °C. Interestingly, on the other hand, the Co dissolution signal reacts stronger to stepping the potential from 4.0 to 5.0 V vs Li^+^/Li, during the conditioning step with flushed EFC, than Mn. Increasing the temperature to 50 and 80 °C profoundly exacerbates TM dissolution as well. This is made clear when stepping the cathode potential from 3.0 up to 4.0 V vs Li^+^/Li. However, the dissolution profiles react on this potential step only sluggishly at 25 °C and moderately at 50 °C; a pronounced peak is observable at 80 °C, especially for the Mn ions. Second, by calculating the TDAs and mass fractions of active transition metals after the bypass accumulation period, the impact becomes even more apparent, as shown in Figure 4.

In the stable voltage window for LIB cathodes between 3.0 and 4.0 V vs Li^+^/Li, the dissolved weight percentage of Mn ions does not rise significantly when increasing the cathode temperature from 25 to 50 °C, and Co dissolution seems to be completely unaffected. During high voltage polarization at 5.0 V vs Li^+^/Li, however, the fractions of all transition metals are largely increased at 50 and 80 °C, respectively. As extended grinding and particle cracking occur at high voltages above ca. 4.2 V vs Li^+^/Li due to changes in the crystal structure,^25−27^ the structural damage might also facilitate dissolution during thermal treatment. This behavior is likely due to exacerbated release of reactive oxygen, which decomposes the (surface) electrolyte and generates acidic species.^21,23,28−31^ Higher temperatures inadvertently increase oxygen emissions from the crystal structure,^32^ which then might produce larger quantities of leaching chemicals. As oxygen plays a vital role in the coordination of transition metals in layered TM oxide structures, its loss has also been connected to TM migration and phase disordering.^33^ It seems reasonable to surmise that an increase in oxygen vacancies in the crystal lattice destabilizes TM ions and facilitates dissolution in the electrolyte as well. In this context, it is interesting to note that Ni has the highest weight fraction of dissolved metals at high temperature and high voltage conditions, although Mn is most susceptible to dissolve at all other conditions in our experiment. High Ni-contents in NCM materials are indeed attributed to a decreased thermodynamic stability,^29^ and increased migration tendency during cycling has been reported for Ni and Co in comparison to Mn.^33^ A high operating voltage, as in the last configuration of the bypass experiment, would therefore create a large number of oxygen vacancies from the crystal structure and induce TM migration. Likewise, the removed oxygen species react with electrolyte molecules to form acidic compounds, which facilitate leaching of the already destabilized metal ions. Elevated temperatures, in addition to increasing oxygen emissions, will likely also accelerate the chemical reactions due to faster kinetics. In summary, increased TM dissolution at high voltages and temperatures, preferentially for Ni and Co ions, is in agreement with literature reports; however, the high dissolution of Mn in this setup for moderate conditions may not be explained in this fashion.

The tendency for Mn dissolution may be linked to research focusing on high-voltage Mn spinel compounds, which also suffer from large Mn dissolution during cycling.^10,11^ It has been demonstrated that the layered TM oxide structure of LIB cathodes experiences structural transition to spinel and rock salt crystal structures during cycling, in which Mn is most prone to dissolve, due to characteristic Jahn–Teller distortions of the trivalent Mn ion.^34^ The surface of the thin-film wafer may have been already disordered, probably due to prolonged storage in the glovebox, which could explain the large difference between Mn and Co dissolution, although Co is usually considered to be less stable than Mn in the layered structure.^33^ At most, several literature reports find approximately stoichiometric dissolution for NCM materials, which in the case of the NCM111, results in similar amounts of dissolved transition metals.^35−37^ The consequences of Mn dissolution for battery systems are remarkable. Studies report that the deposition of dissolved Mn on the anode, even more than for Co and Ni, leads to detrimental SEI thickening, Li ions capture, and overall capacity loss of the battery.^37,38^ It has also been shown that Mn deposits more readily at the anode side and that elevated temperatures exacerbate this tendency. Buchberger et al. found twice the amount of Mn compared to Ni and Co on aged graphite electrodes after extended cycling at 25 °C at two different cutoff voltages (4.2 and 4.6 V vs Li^+^/Li). At 60 °C, however, the amounts of Ni and Co on the anodes remained almost constant, but the Mn content was six times higher, shifting the ratio to 10:1.^39^ This leads to a significant increase in the cell impedance, and hence a reduction in capacity and battery lifetime. Our results show that overall TM dissolution is increased for LIBs frequently operating at elevated temperatures. Although our results are not entirely applicable to full cells, due to considerable changes in components and dimensions, they still highlight the importance of temperature control for TM dissolution and, thus, battery performance as a whole.

Although the bypass experiments shine light on the dissolution behavior of the TMs at moderate conditions, the concurrent heating of the electrolyte in the flow cell disables focusing on studying the interfacial processes of TM dissolution. As described previously, an immobile layer of electrolyte will remain on the cathode surface at all times and reach into the bulk electrolyte for stagnant conditions. Employing constant electrolyte flow through the EFC, however, creates a hydrodynamically steady-state electrolyte layer with lateral velocity approaching 0. In a comparable V-shaped flow cell with unobstructed flow channels, this layer has an approximate thickness of 50 μm, according to simulation.^24^ In a real cell, with two additional electrodes in closest proximity to the working electrode surface, it is reasonable to assume that local flow regimes will be more turbulent and hence the electrolyte surface layer even thinner. For all of these conditions, heat transfer is sufficient to temper the entire cathode surface–electrolyte interface layer essentially at once, so that the cathode surface and electrolyte layer can be treated as having the same temperature (SI). Due to the otherwise fast replacement of liquid cell volume, we estimate that bulk electrolyte temperature will not exceed 34 °C, when the cathode is kept at 60 °C (see the SI). As the flowing electrolyte immediately cools down after passing the working electrode, it is reasonable to surmise that no significant additional decomposition takes place. Based on the literature results presented above, dissolution phenomena are then exclusively governed by temperature effects at the cathode surface and in the surface electrolyte layer.

**Figure 5:**
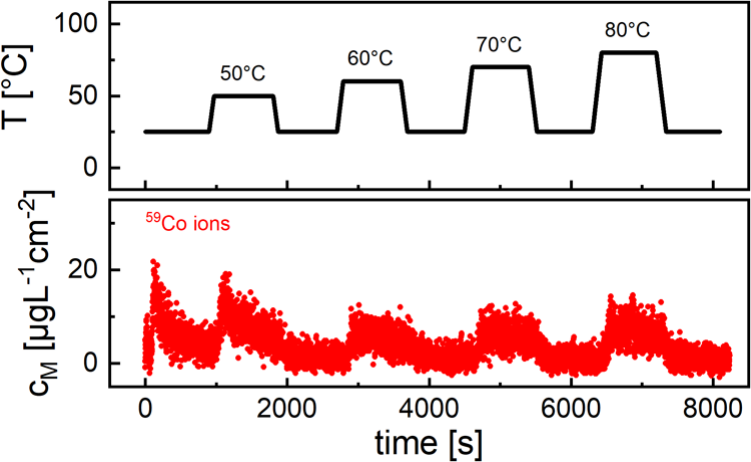
Dissolution of Co ions from thin-film NCM111 cathode during constant polarization at 4.7 V vs Li^+^/Li and different temperatures. At *t* = 0, the potential was stepped to 4.7 V.

Building on the constant voltage/constant temperature experiments carried out in bypass configuration, we performed an additional set of measurements with electrolyte flow; however, this time, the temperature of the cathode was modified during polarization, and temperature and dissolution transients were recorded. A high voltage of 4.7 V vs Li^+^/Li was chosen because, at this potential, baseline dissolution is discernible from blank electrolyte values for the first time. At 25 °C and constant electrolyte flow, the potential of the cathode was stepped to 4.7 V, and after 15 min, the temperature was ramped up to 50 °C as fast as possible and maintained. After 15 min, the cathode was cooled down to 25 °C again. This procedure was repeated with 60, 70, and 80 °C terminal temperatures, respectively. The average heating/cooling rate of the setup was about 0.4 K/s. Figure 5 displays the temperature and dissolution plots; note that only Co dissolution is detectable for these operating conditions.

It is clearly visible that the dissolution signal increases in response to the elevated cathode/surface electrolyte temperatures; surprisingly, the reaction is not uniform for the entire temperature program. When applying the first temperature ramp to 50 °C, Co dissolution increases very swiftly, approximately 20 seconds after the ramp had been started, at a temperature onset of ca. 30 °C. After reaching a peak value, dissolution slowly declines for the remainder of the temperature hold; the end dissolution level is only slightly higher than the baseline. For 60, 70, and 80 °C, dissolution rises considerably later with a delay of ca. 60 s and onset temperatures around 50 °C (See Figure S6 for determination of onset). In addition, the maximum dissolution levels are lower compared to the 50 °C peak but stay constant for the entire hold. The average dissolution levels also increase modestly by 5% for 70 °C and 14% for 80 °C compared to the 60 °C plateau. Unfortunately, we cannot provide a comprehensive explanation for this phenomenon, in part due to the lack of comparable real-time dissolution studies in the literature. If the dissolution mechanism was only governed by oxygen release and its corresponding phase transitions and electrolyte decomposition at the electrode interface, the dissolution profiles in Figure 5 should all be similar. The potential of the cathode is well above the onset of oxygen emission described by the Gasteiger group,^32^ and both oxygen release rate and interface temperature (hence reaction kinetics with electrolyte) should be constant with regard to the observed time frame of 15 minutes. A decline in the dissolution signal could be explained in the context of this hypothesis by the reduction in oxygen removal or depletion of the electrolyte interface layer, either of which results in decreasing formation of electrolyte decomposition products. As the rate of dissolution is constant for subsequent temperature holds, both arguments seem to be insufficient. The delayed onset of additional (observable) dissolution during the last three temperature ramps, all close to 50 °C, might be an indication of dissolution controlled by electrolyte decomposition products. Second, due to uniform behavior after the first hold at elevated temperature, the structure of the entire cathode electrolyte interface—cathode surface, deposited inorganic and organic compounds, and surface electrolyte layer—may have stabilized during this period. For example, surface impurities such as Li_2_CO_3_ might be decomposed, reacting with the cathode surface and electrolyte to induce dissolution below the expected decomposition temperatures of the electrolyte. The subsequent reduction in the dissolution signal may then be due to depletion of transition metals in the outermost surface regions, followed by slower dissolution from cathode layers located further away. Dissolution from these cathode regions, much more abundant in transition metals, could then be uniform throughout following temperature cycles. To the best of our knowledge, there are no literature reports on this specific topic, given the experimental difficulty of probing cathode electrolyte interfaces and the short time frame experienced in this study. However, decomposition of electrolytes in the presence of transition-metal oxide surfaces and resulting modified surface layers have already been discussed.^22^ The change in dissolution mechanism is another indication of the pivotal role of interfacial properties for a fundamental understanding of battery systems and battery applications.

**Figure 6:**
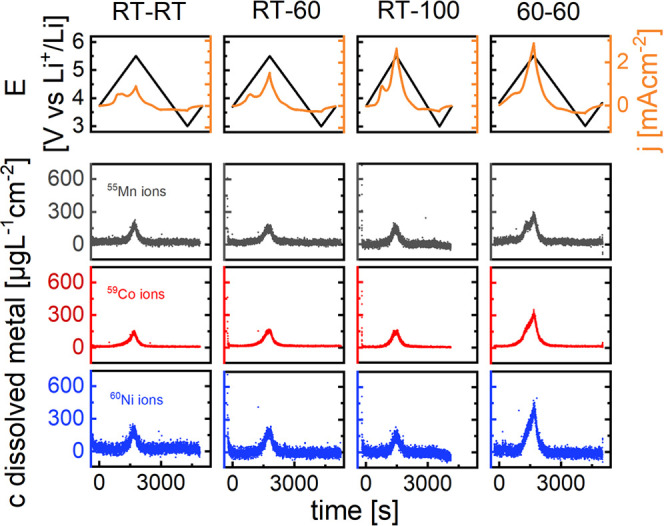
Voltage and current–density time plots with simultaneously measured dissolution profiles during cyclic voltammetry, 3.0–5.5 V vs Li^+^/Li at a scan rate of 1 mV s^–1^. RT-100 was cycled at 1.2 mV s^–1^ due to insufficient electrolyte volume after heat treatment. From left to right, cathode and electrolyte temperatures [°C] are: 25/25, 25/60, 25/100, 60/60.

Literature reports indicate that electrolyte decomposition reactions change in the presence of battery electrodes and that their chemical and structural composition impacts electrolyte stability.^21,23^ In order to study the impact of decomposition products in the electrolyte further, we prepared electrolyte samples preheated at 60 and 100 °C for 18 h in pp-vials and argon atmosphere. It should be noted that the cathode is known to react with electrolytes, especially at high state of charges, so that these electrolyte samples will likely not fully represent the composition of electrolyte decomposed in situ. However, this approach enables us to distinguish which source of decomposition products impacts the dissolution process. Cyclic voltammograms with the thin-film NCM111 samples were performed between 3.0 and 5.5 V vs Li^+^/Li using electrolytes preheated at different temperatures. The scan rate was set to 1 mV s^–1^. Our analysis comprises four combinations with varying working electrode temperatures and preheated electrolytes, as presented in Table 1. The dissolution curves of the experiments are summarized in Figure 6.

**Table 1:** Heating Plate and Electrolyte Temperature Configurations for Operando Flow Cell Experiments

	electrolyte pretreatment temperature		
temperature heating plate [°C]	25	60^a^	100^a^
25	RT-RT	RT-60	RT-100
60		60-60	

a Electrolyte preheated for 18 h in an Ar atmosphere and then used at 25 °C.

The sample with the electrolyte preheated at 100 °C (RT-100) had to be cycled with 1.2 mV s^–1^ because of evaporation losses. With the thin-film cathodes used in this experiment, a higher scan rate results in a higher metal dissolution. Thus, the dissolution values presented in Figure 6 are slightly higher than they would have been at 1 mV s^–1^.

Cycling with the electrolytes preheated for 18 h at either 60 °C (RT-60) or 100 °C (RT-100) does not change the NCM111 dissolution profiles significantly, compared to the reference measurement without electrolyte pretreatment (RT-RT). This is quite surprising, as the oxidation current is much higher for the preheated electrolyte samples. The peaks appear to be slightly more leveled, but no impact on either height or position is apparent. While the electrolyte pretreated at 60 °C did not decompose according to our GC-MS analysis, the electrolyte pretreated at 100 °C did (Figures S7 and S8). The color of the electrolyte turned into a vibrant red, and a considerable portion of the 50 mL sample evaporated (Figure S9), despite closing the plastic vessels firmly. Metal vessels were considered but not used due to the possibility of contamination with metal ions. The reduction of solvent volume inadvertently changes the concentration of LiPF_6_. In addition, the decomposition of the salt also occurs at a much higher rate. GC-MS analysis (Figure S8) indicates that EMC as the lower boiling solvent (boiling point 107 °C compared to 243 °C of EC) was affected in particular, generating a large variety of low and high mass decomposition products. Nevertheless, we assume that the voltammetric results are comparable to the usual electrolyte, as the cell impedance did not change significantly with the corresponding electrolyte sample. Surprisingly, even the significantly aged electrolyte sample does not increase the dissolution overall, as shown in Figure 6. This is in contrast to decomposition products from addition of water to the electrolyte, which we have shown in our previous publication.^17^

**Figure 7:**
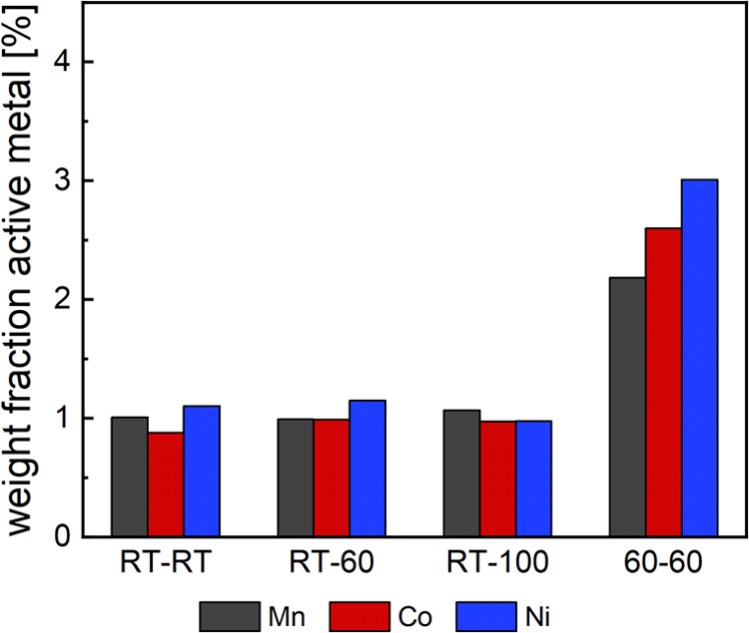
Dissolved percentage of total active metal of Mn, Co, and Ni ions after CVs of 3.0–5.5 V vs Li^+^/Li at a scan rate of 1 mV s^–1^ (1.2 for RT-100). From left to right, heating plate and electrolyte temperatures [°C] are 25/25, 25/60, 25/100, 60/60.

When increasing the temperature of the cathode film (heated plate) to 60 °C, dissolution levels increase markedly. The peak concentrations of Mn and Ni rise from ca. 180 to 250 and 380 μg L^–1^ cm^–2^, respectively, while for Co, an increase from 140 to 300 μg L^–1^ cm^–2^ is found. The oxidation current at the vertex potential of 5.5 V is the highest among all CV experiments in this work, whereas the first oxidation peak at 4.6 V is comparable to the reference measurement at 25 °C and even lower than with the electrolyte preheated at 100 °C. As dissolution is much higher at the same time, a bigger fraction of the current seems to be directed toward the dissolution process, compared with the heated electrolyte samples. It is also noticeable that the shape of the dissolution peaks differs from the previous curves. Dissolution at 25 °C cathode temperature generates highly symmetrical peaks, which align with the vertex potential; the scan at 60 °C, however, incurs two sections at the front of the peak, which align with the current signal peaks at 4.6 and 5.5 V. During the backscan, the dissolution signal returns much quicker to baseline level, which results in a rather fronted curve. The corresponding mass fractions of the total dissolved active material are presented in Figure 7.

As noticed before with the bypass experiments, Ni dissolution seems to be most affected by the increased cathode temperature, in particular at high operating voltage. In contrast, Mn dissolution is significantly reduced in this setup by 4 and 7 ng compared to Co and Ni TDAs (−22 and −33%). It is worth noting that a precise estimation of the Mn dissolution onset and, thus, the integration value, is difficult due to the rather high standard deviation of the Mn signal. However, the same error applies to Ni dissolution as well, which suggests that the difference in dissolution is indeed significant. With regard to the TDAs at room temperature, on the other hand, this might also indicate that preferential dissolution of Mn over Co is significant as well, in alignment with Jahn–Teller distortions of Mn spinels and with the bypass experiments presented above. As explained previously, high temperature and high voltage conditions intensify Co and Ni dissolution, due to preferential migration and concomitant oxygen release, which decrease the thermodynamic stability of the material.^40,41^ In addition, the Ni^3+^/Ni^4+^ redox couple is the main electrochemically active species in the NCM cell reaction,^42^ supported by the Co redox couple at higher potentials. This also explains why approximately stoichiometric amounts of Mn, Co, and Ni are found when the cathode is cycled at room temperature (with Mn and Ni being slightly less stable than Co), while TM dissolution follows the order Mn < Co < Ni at 60 °C working temperature.

Coming back to the question of the contributions of electrolyte decomposition products to the dissolution process, it is clear that higher cathode temperatures exacerbate TM dissolution, whereas even strongly decomposed electrolytes do not affect TM dissolution on their own in our experiments. It has to be noted that the RT-100 electrolyte sample, in particular, lacks gaseous components, which would have been present in closed lithium-ion batteries—and therefore might participate in the dissolution process.^43^ Campion et al., for example, have proposed an autocatalytic decomposition cycle for EMC, which consumes PF_5_, a volatile decomposition product of the conductive salt LiPF_6_.^7^ On the other hand, the presence of electrolyte surface layers, which are presumably subject to the same temperature as the cathode surface, also rationalizes the formation of novel decomposition products and modification of the cathode surface.^20^ Our findings concerning transition-metal dissolution may be explained with mechanisms described in the literature, for example, the preferential dissolution of Ni and Co at high temperatures and high state of charge. High dissolution of Mn at moderate operating conditions in bypass configuration, however, and change in dissolution behavior during the initially applied temperature ramp in open configuration do not fit with electrolyte decomposition as the only driving force for TM dissolution. In both cases, the structure of the cathode electrolyte interface may play a vital role in the dissolution mechanism, for example, surface reconstruction and Mn spinel dissolution behavior; hence, a final evaluation of the impact of electrolyte heating on TM dissolution is difficult. In summary, our findings suggest that dissolution processes are more nuanced than usually described in literature, so that real-time dissolution analysis could be a valuable addition to interfacial studies.

## Conclusions

By employing a bypass in a flow cell setup, we have demonstrated that significant TM dissolution may occur even at slightly elevated temperatures such as 50 °C and moderate voltages of 4.0 V vs Li^+^/Li. For these conditions, Mn has been particularly susceptible to dissolve, whereas we could not find increased dissolution of Co and Ni. The Mn dissolution behavior may be related to phase transitions of the layered material to Mn spinels, which are known to degrade readily, but we did not investigate structural changes in our sample. These results suggest that oxygen release and electrolyte decomposition are not the only driving forces of dissolution because these would likely affect Ni and Co as well. At even higher temperature and voltage conditions, 80 °C and 5.0 V vs Li^+^/Li, Ni dissolution was predominant, and the increase in Co dissolution was much more pronounced than for Mn. This is in accordance with literature reports on the individual stability of transition-metal ions in NCM bulk materials. By applying temperature transients during constant polarization, we found fast dissolution kinetics at only slightly increased temperature for the first applied ramp, whereas dissolution set in much later during subsequent ramps with the same sample. The change in dissolution mechanism might indicate an alteration of the cathode electrolyte interface, but a comprehensive explanation could not be provided. Performing cyclic voltammetry with thermally aged electrolytes in operando mode of the EFC, we found no increased dissolution. Raising the cathode temperature to 60 °C, however, exacerbated TM dissolution immediately. The increase in dissolution may be explained with enhanced oxygen release and decomposition kinetics in the surface electrolyte layer as proposed in the literature. We also find that Ni and Co dissolve more easily than Mn in this experiment, which is also consistent with the individual migration tendencies in NCM bulk materials.

## Methods

### Chemicals

Electrolytes with 1.0 mol L^–1^ LiPF_6_ in a binary mixture of carbonate solvents EC/EMC 3:7 (w/w) were directly purchased from E-Lyte Innovations GmbH, Münster, introduced to the glovebox, and used as received. The 1% (w/w) nitric acid solution for dilution of the electrolyte feed to the ICP-MS was prepared from concentrated 68% (w/w) nitric acid (ULTREX II, J.T. Baker) and ultrapure water (Merck Millipore). Calibration standards and internal standard solution (300 μg L^–1^ Ge in 1% HNO_3_) were prepared from 1000 mg L^–1^ metal salt solutions (Certipur, Merck) of Ge, Ni, Mn, and Co.

### Cathode Manufacturing

A thin-film NCM111 layer, with a nominal thickness of 100 nm, was spin-coated onto a 100 nm Pt/c-sapphire (Al_2_O_3_) wafer. Details of the spin-coating process are described elsewhere.^18^ The thin-film sample was chosen due to its phase-pure, additive-free structure, which provides ideal properties for mechanistic dissolution studies. Samples were packed and stored under inert conditions (Ar-filled glovebox atmosphere). Nominal loading of active material is 80 μg cm^–2^ (corresponding to 3.4 μg of active material; and 0.69 μg of Ni and Co, and 0.65 μg of Mn, respectively).

### Experimental Setup

The setup adapted from Wachs et al.^15^ and Ranninger et al.^16^ has been described in our previous publication.^17^ The translational stage (Physik Instrumente PI) was set to different temperatures (25, 50, 60, 70, and 80 °C in this work), with the help of a homemade Peltier-controlled electrode holder platform. Both the cathode and the electroanalytical flow cell were pressed on top. We also included an adjustable three-way stopcock and a bypass tubing (Figure S1).^44^ By switching the lever on the valve, either the EFC or the bypass tube is flushed by the electrolyte, which allows us to perform stagnant collection experiments with the EFC. Stagnant electrolyte operating conditions in the EFC will hence be called “bypass configuration,” whereas the opposite is called “EFC configuration.” Measurement conditions for the ICP-MS have been described elsewhere.^15^

### Electrochemical Measurements

The experiments were performed with a VSP-300 potentiostat (BioLogic, France). Prior to each experiment, the internal cell resistance (cathode interface and electrolyte) was determined using impedance spectroscopy, yielding 100–400 Ω. Due to the small currents registering well below 100 μA, IR-compensation was omitted. Polarization experiments with constant potentials in bypass configuration were conducted at first, keeping the cathode at 3, 4, and 5 V vs Li^+^/Li, respectively. Second, we measured online transition-metal dissolution during constant polarization at 4.7 V vs Li+/Li and transient temperature. In the last set of experiments, cyclic voltammograms (CV) were performed in the range of 3.0–5.5 V vs Li^+^/Li, with a scan rate of 1 mV s^–1^. As stated in our previous paper,^17^ these scan rates result in a very high effective cycling rate of ca. 2C. However, in our setup, we did not find major deviations in the dissolution process compared to cycling rates below 1C.

## Supporting Information Available

Heat-transfer estimation in EFC; bypass configuration for accumulation experiments; ghost peaks created by the bypass setup; Ni dissolution in bypass configuration during constant voltage experiments; determination of onset temperature for dissolution during temperature ramp; GC-MS analysis of preheated electrolytes; and calibration of the reference electrode (PDF)

## Abbreviations

LIB: lithium-ion battery
EFC: electroanalytical flow cell
ICP-MS: inductively coupled plasma mass spectrometry
TM: transition metal
EV: electric vehicle
CAM: cathode active material
SEI: solid electrolyte interface
EFC: electroanalytical flow cell
NCM: lithium nickel-cobalt-manganese oxide LiNi*_x_*Co*_y_*Mn*_z_*O_2_ (*x* + *y* + *z* = 1)
CV: cyclic voltammetry
TDA: total dissolved amount
HP: heating plate
OCV: open-circuit voltage
