# Supplementary material for: Contribution of Electrolyte Decomposition Products and the Effect of Temperature on the Dissolution of Transition Metals from Cathode Materials
Source: ACS Omega. 2023 Aug 25;8(36):32606–14. doi: 10.1021/acsomega.3c03173 (PMC10500674; doi:10.1021/acsomega.3c03173)
Supplement: Supplementary file 1 — ao3c03173_si_001.pdf [file ao3c03173_si_001.pdf]

Supporting Information

The Contribution of Electrolyte Decomposition Products and the Effect of Temperature on the Dissolution of Transition Metals from Cathode Materials

Janik Lüchtefeld[a*], Ming Yu Lee[a], Hendrik Hemmelmann[†], Susanne Wachs[a≠], Christopher Behling[a], Karl J. J. Mayrhofer[a], Matthias T. Elm[†‡], Balázs B. Berkes[*]

[a]Helmholtz Institute Erlangen-Nürnberg for Renewable Energy (IEK-11), Forschungszentrum Jülich, Cauerstr. 1, 91058 Erlangen, Germany

[a]Department of Chemical and Biological Engineering, Friedrich-Alexander-Universität Erlangen-Nürnberg, Cauerstr. 1, 91058 Erlangen, Germany

[†]Center for Materials Research, Justus-Liebig-University Gießen, Heinrich-Buff-Ring 16, 35392 Gießen, Germany

[‡]Institute of Physical Chemistry, Justus-Liebig-University Giessen, Heinrich-Buff-Ring 17; Institute of Experimental Physics I, Justus-Liebig-University Giessen, Heinrich-Buff-Ring 16, 35392 Giessen, Germany

‡BASF SE, Carl-Bosch-Str. 38, 67056 Ludwigshafen am Rhein, Germany

Corresponding Author

Helmholtz Institute Erlangen-Nürnberg for Renewable Energy (IEK-11), Forschungszentrum Jülich, Cauerstr. 1, 91058 Erlangen, Germany, Email: j.luechtefeld@fz-juelich.de

Table of content

Heat-transfer estimation in EFC 3
Bypass configuration for accumulation experiments 5
Ghost peaks created by the bypass setup 5
Ni dissolution in bypass configuration during constant voltage experiments 6
Determination of onset temperature for dissolution during temperature ramp 7
GC-MS analysis of pre-heated electrolytes 9
Calibration of the reference electrode 11
References 11

Heat-transfer estimation in EFC

The heat transfer from the heated cathode surface to the electrolyte was estimated with the model of a laterally flushed plane at constant temperature[1]. For a laminar interface layer the average Nusselt number Nu is

$$Nu = 0.664\sqrt{Re}\sqrt[3]{Pr} \qquad S1$$

With Re and Pr being the Reynolds number and Prandtl number, respectively:

$$Re = \frac{w \cdot l}{v} \qquad S2$$

$$Pr = \frac{\eta \cdot c_p}{\lambda} \qquad S3$$

w is the electrolyte velocity, l is the characteristic length (in this case the diameter of the cathode); ν is the kinematic viscosity, η is the dynamic viscosity, c_p is the specific heat, and λ is the thermal conductivity of the electrolyte. Table S1 summarizes the corresponding properties:

Table S1: characteristic properties for heat transfer estimation

Physical properties of electrolyte and cathode setup		
$\dot{V}$	150	µL min^{-1}
A	4.02	mm^2
w	0.6217	$10^{-3} \cdot$ m s^{-1}
l	2.25	mm
v	2.107[a]	$10^{-6} \cdot$ m^2 s^{-1}
η	0.0025[2]	kg m^{-1}s^{-1}
c_p	2000[3]	J kg^{-1}K^{-1}
λ	0.6[3]	W m^{-1}K^{-1}

[a] has been calculated from η and density $\rho = 1186.54\ kgm^3$, with $v = \frac{\eta}{\varrho}$

According to equation S1, $Nu = 1.551$; variation of physical properties of the electrolyte with temperature have not been included. Nu is defined as

$$Nu = \frac{\alpha \cdot l}{\lambda} \qquad S4$$

Where α is the heat transfer coefficient from the cathode to the electrolyte. It is

$$\dot{\text{Heat}} = \dot{m} c_p \Delta T = \alpha A \Delta T_{\log} \qquad S5$$

$\dot{m} = \dot{V}/\rho$ is the mass flux of electrolyte (3 mg s^{-1}), $\Delta T = T_{\text{LP57,out}} - T_{\text{LP57,in}}$ is the temperature difference of the electrolyte, A is the geometric surface area of the cathode (table S1), $\Delta T_{\log}$ is the logarithmic temperature difference between electrolyte entering and exiting the cathode surface:

$$\Delta T_{\log} = \frac{(T_{\text{cathode}} - T_{\text{LP57,in}}) - (T_{\text{cathode}} - T_{\text{LP57,out}})}{\ln\left(\frac{T_{\text{cathode}} - T_{\text{LP57,in}}}{T_{\text{cathode}} - T_{\text{LP57,out}}}\right)} \qquad S6$$

By choosing a temperature for $T_{\text{LP57,out}}$ a second value for α may be calculated and compared to the one obtained by the Nu number. For a volume flux of 150 µLmin^{-1} and an average cross-sectional area over the cathode of 4.02 10^{-6} m^2, the calculated α from eq. S4 is 414 W/m^{2}K. This corresponds to a terminal temperature of the electrolyte of 34 °C.

In a similar way, the heat transfer to the cathode wafer and electrolyte surface layer can be estimated. The sample consists of a 500 µm Al_2O_3 (c-sapphire) layer, sputtered on both sides with 100 nm Pt and the final 100 nm NCM111 layer. Due to its thickness s, the sapphire wafer is the only meaningful contributor to the overall thermal conductivity, which is approximately 38 W/mK in the temperature range 25 to 50°C. This value has been derived as the average from the thermal conductivity at 0°C (46.06 W/mK) and 100°C (25.12 W/mK), data supplied by the vendor MTI Corporation. Further geometric and thermal properties of the wafer are its Area A 0.00203 m^2, mass m 0.00406 kg, and the specific heat c_p of ca. 1000 J/kgK. Considering a constant temperature of the heating plate T_P and constant surface temperature of the wafer T for a given time differential, the heat flux may be described similar to equation S5:

$$mc_p\frac{dT}{dt} = \frac{\lambda A}{s}(T_P - T) \qquad \text{S7}$$

Integration of S6 and solving for T yields an expression for the wafer surface temperature at any given time:

$$T = T_P - (T_P - T)e^{-\frac{\lambda A}{mc_p s}t} \qquad \text{S8}$$

The mass of the heating plate is much larger than the mass of the wafer, so T_P will not be affected largely by the wafer itself. With the values given above, T equals T_P in much less than a second (in the range of 10 ms), so heating of the wafer may be treated as instantaneous in first approximation. For consideration of the electrolyte surface layer, the layer thickness δ was estimated from numerical simulations of the flow velocity profile in the flow cell. For laminar conditions, δ is in the range of 10 to 10^2 µm; the film thickness may also be approximated as the ratio of thermal conductivity λ of the electrolyte, and heat transfer α from cathode to electrolyte:

$$\delta \approx \frac{\lambda}{\alpha} \qquad \text{S9}$$

For the battery electrolyte and given δ, α is in the range of 10^3 to 10^4 W/m^2K, which also signifies adaptation of the cathode surface temperature in less than one second (in the range of 10 µs). As an estimation, cathode surface and surface electrolyte layer have the same temperature, which is the temperature of the heating plate.

Bypass configuration for accumulation experiments

The schematic bypass setup is shown in figure S1.

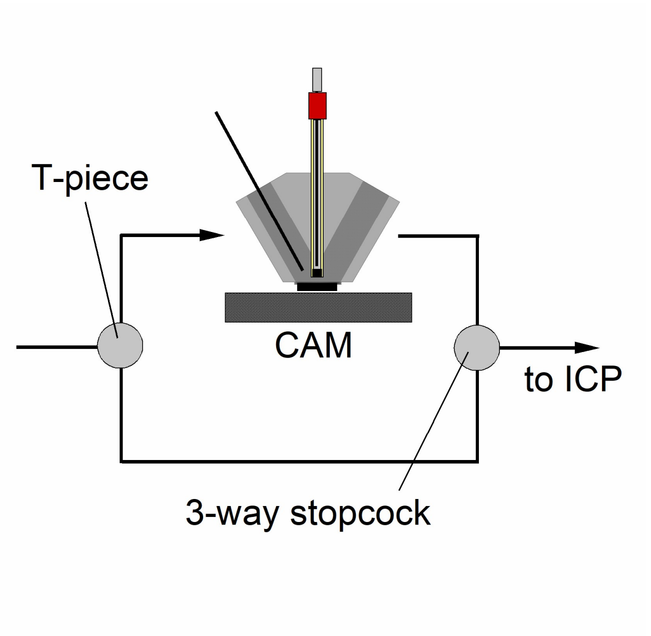

Figure S1. Schematic of the bypass used in the corresponding discontinuous experiments.

Ghost peaks created by the bypass setup

Due to different pressure losses along the bypass tubing and the EFC, the mass transport of analytes and internal standard Ge into the ICP-MS is disturbed: Switching from EFC to bypass yields a rise in the analyte signal, switching back corresponds to a marked drop. Figure S2 illustrates the effect with baseline dissolution of TMs in the electrolyte.

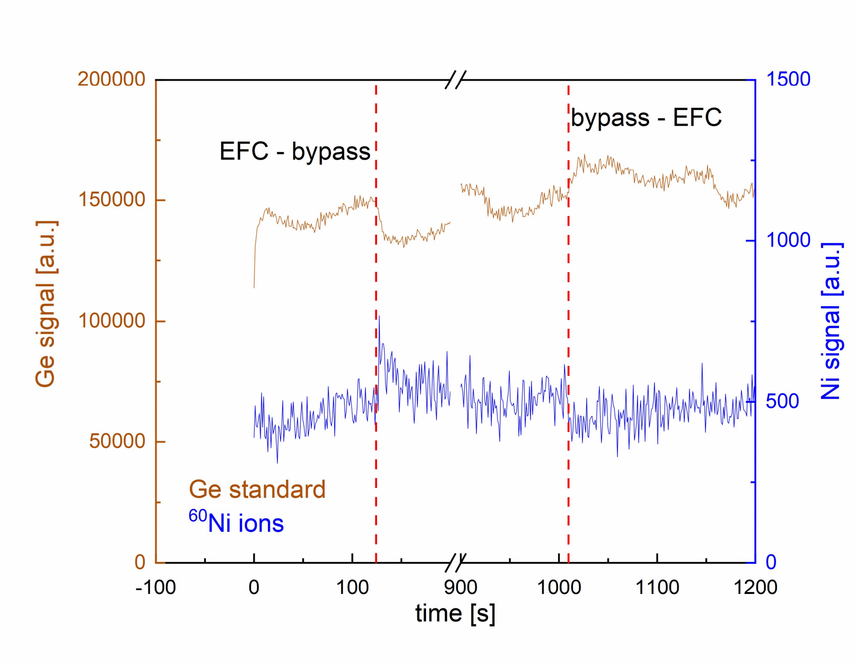

Figure S2. Analyte signal peaks created by switching between bypass tubing and EFC.

Ni dissolution in bypass configuration during constant voltage experiments

Ni dissolution could not be detected for the majority of constant potential dissolution experiments due to its increased baseline and signal fluctuations. The graphs of the Ni signal for dissolution at 25, 50, and 80°C and corresponding potentials of 3.0, 4.0 and 5.0 V vs Li^+/Li are presented in figures S3 – S5.

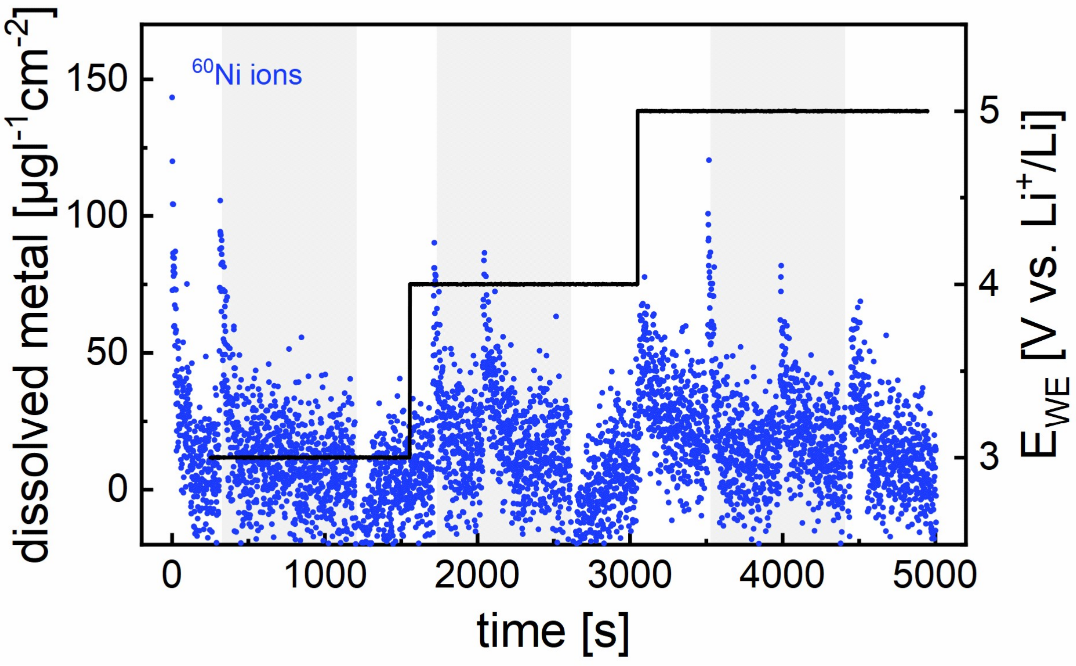

Figure S3: Dissolution profiles of Ni at 25°C in an EFC with stagnant electrolyte at 3.0, 4.0, and 5.0 V vs Li^+/Li. After stepping up the applied potential, the EFC was flushed with electrolyte until constant dissolution values were achieved. Then, the cell was conditioned 15 minutes with active bypass, before electrolyte was directed through the cell again.

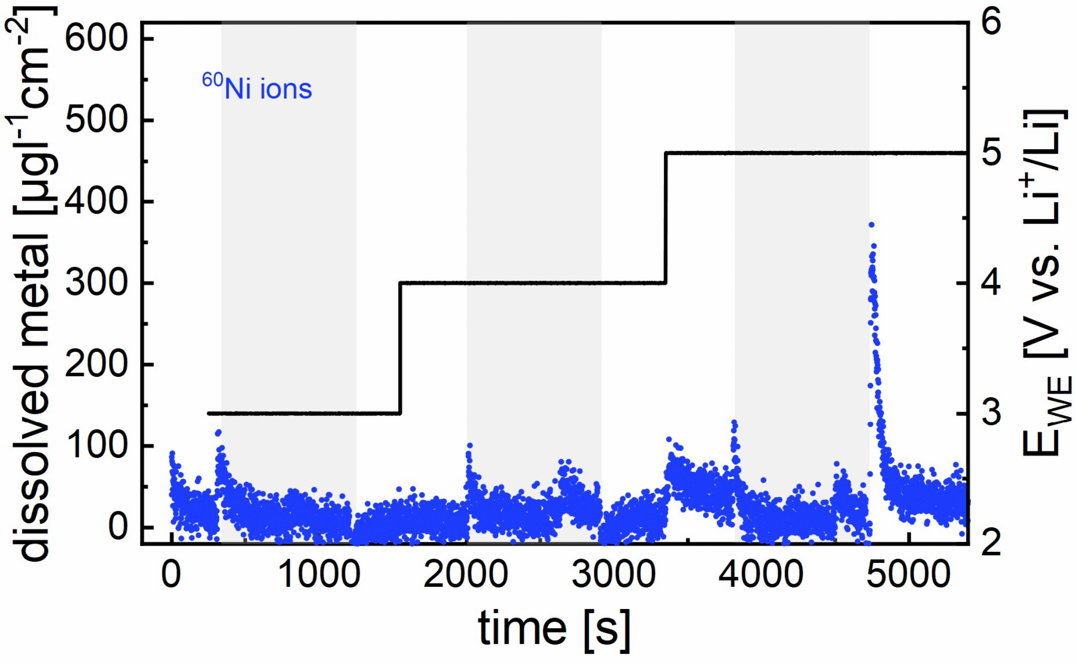

Figure S4: Dissolution profiles of Ni at 50°C in an EFC with stagnant electrolyte at 3.0, 4.0, and 5.0 V vs Li^+/Li. After stepping up the applied potential, the EFC was flushed with electrolyte until constant dissolution values were achieved. Then, the cell was conditioned 15 minutes with active bypass, before electrolyte was directed through the cell again.

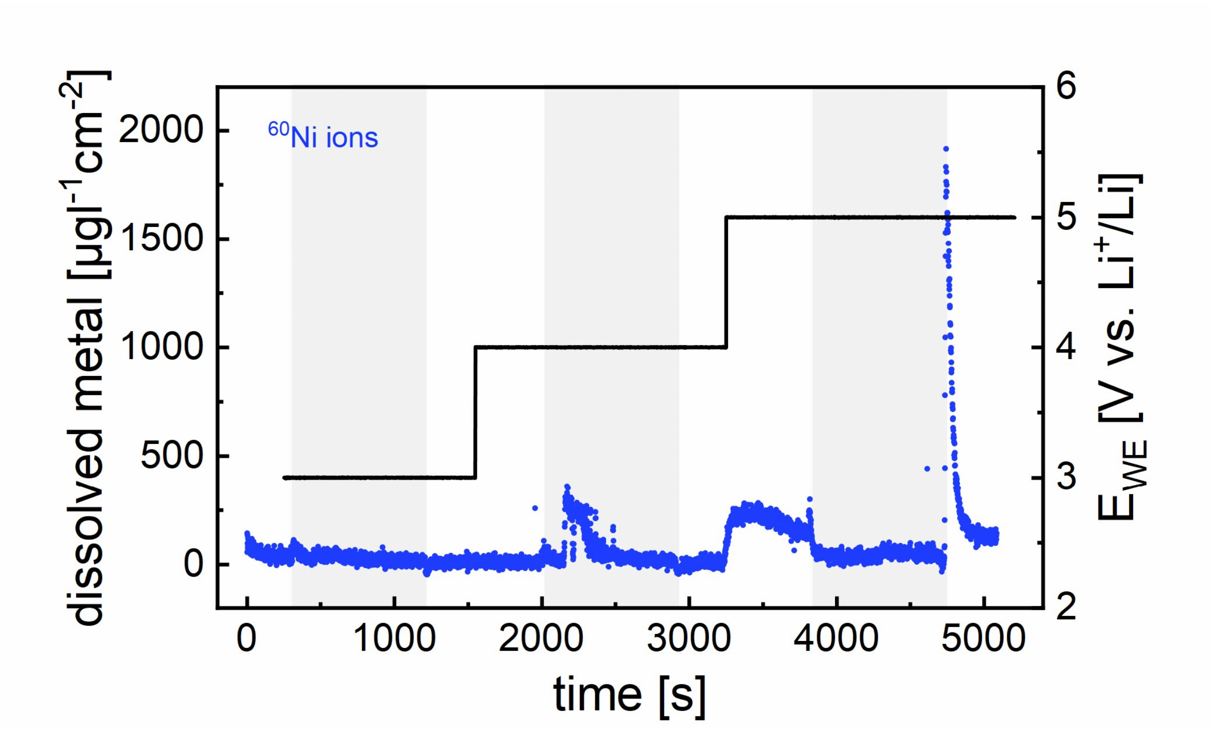

Figure S5: Dissolution profiles of Ni at 80°C in an EFC with stagnant electrolyte at 3.0, 4.0, and 5.0 V vs Li^+/Li. After stepping up the applied potential, the EFC was flushed with electrolyte until constant dissolution values were achieved. Then, the cell was conditioned 15 minutes with active bypass, before electrolyte was directed through the cell again.

Determination of onset temperature for dissolution during temperature ramp

Applying a temperature ramp during constant polarization at high voltages (i.e. 4.7 V vs Li^+/Li) results in increased dissolution of transition metals from the cathode. Due to the superior sensitivity of the ICP-MS to Co-dissolution, the transients of the dissolution process may be studied with high resolution. In order to determine the (temperature) onset of dissolution, the Co baseline before the temperature ramp and the subsequent dissolution transient were fitted by linear functions in the OriginPro 2020 software. The intercept of both functions (t_i) was compared with the temperature-time data and hence the onset temperature acquired. Figure S6 displays the combined Co dissolution and temperature data, as well as the fitted linear functions.

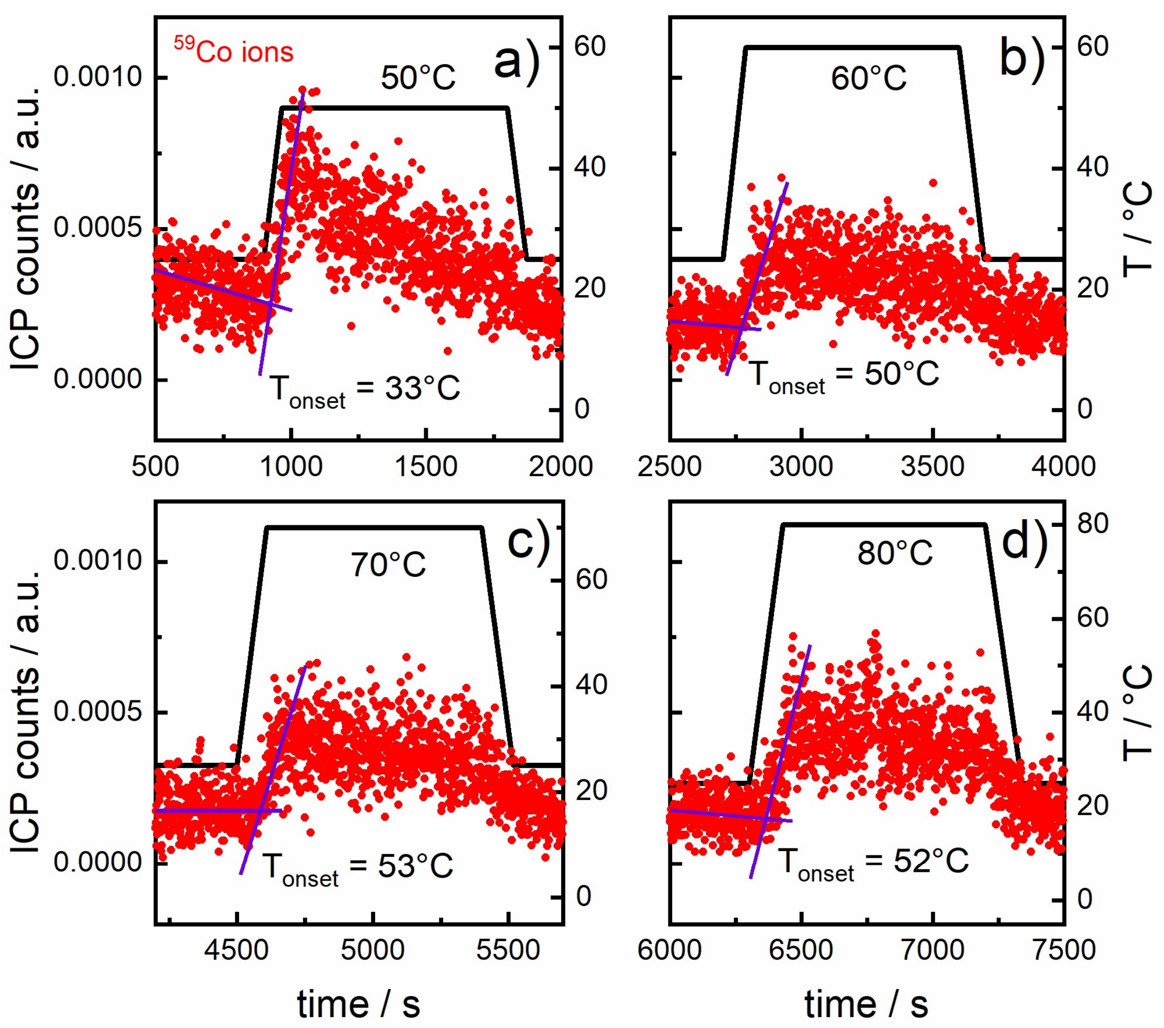

Figure S6. Combined dissolution and temperature data for temperature ramps to specified temperature during constant polarization of NCM111 thin-film cathode at 4.7 V vs Li^+/Li. Mn and Ni signals have been omitted due to insufficient response in ICP-MS. The purple lines have been fitted to the Co baselines and dissolution during the temperature transients, where the intercepts signify the onset of increased dissolution. Terminal temperatures are a) 50°C, b) 60°C, c) 70°C, d) 80°C. The temperature ramps have been applied in the order of the individual diagrams a) to d), with 15 minute intervals at 25°C in between.

Clearly, the temperature onset during the first ramp is much lower compared to the subsequent transients. Also, the slope of the dissolution response is higher (approximately twice as high). It has to be noted that, although the overall sensitivity of the ICP-MS system is very high towards Co, inline dilution[4] by the factor 1:49 of the electrolyte flow prior to injection increases the effective baseline considerably. Therefore, an appreciable amount of Co will have already dissolved before the indicated onset temperatures.

GC-MS analysis of pre-heated electrolytes

An electrolyte sample (LP57, ELyte) was put insight a plastic syringe and heated for 18 hours at 60°C in an Ar-filled oven (slight underpressure of 100 mbar applied). As reference, an electrolyte sample from the glovebox was taken as well. The samples were injected directly into the measuring device (Agilent 5977C GC/MSD) without prior dilution and separated on a HP-5ms Ultra Inert column (Agilent). The oven temperature was increased from 55°C to 90°C with a 12°C/min heating rate and subsequently ramped to 300°C with 40°C/min, at which the temperature was maintained for 4 minutes. The total ion chromatograms are displayed in fig. S7.

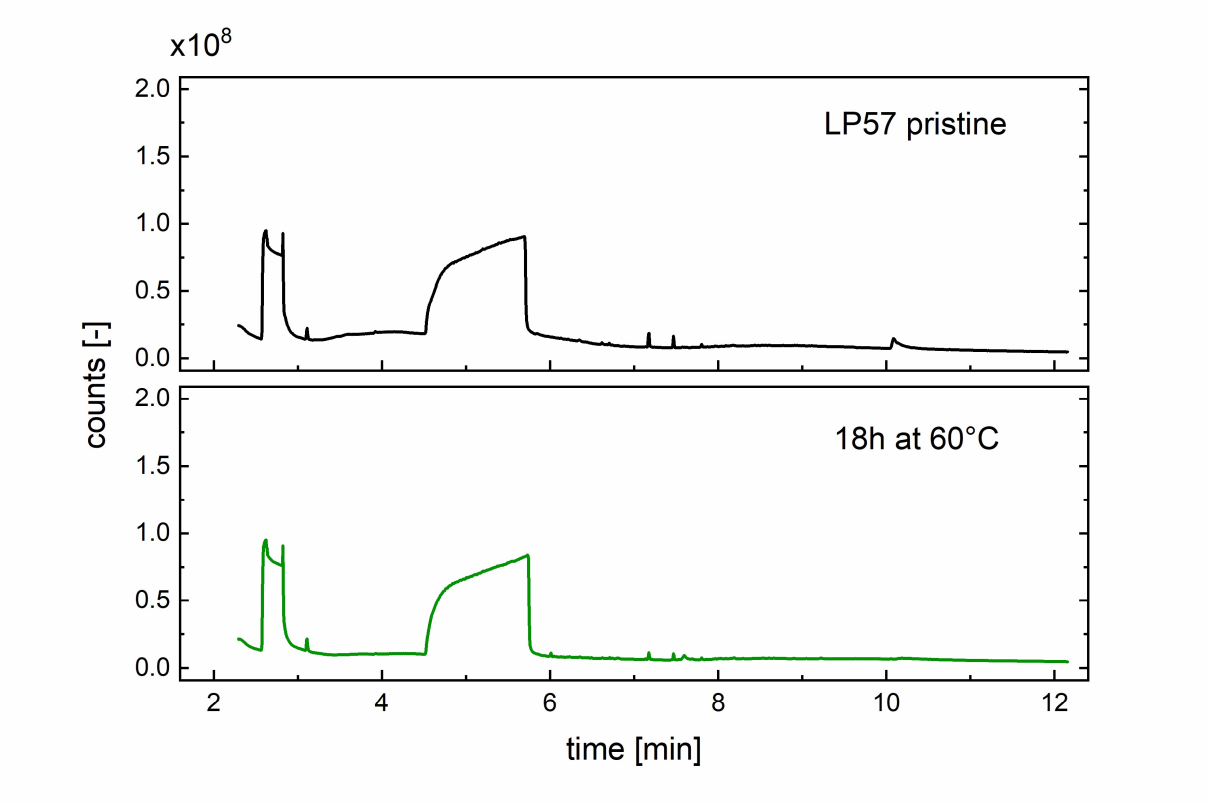

Figure S7. Total ion chromatograms (TIC) of LP57 electrolyte without and with pre-heating at 60°C for 18h in an Ar-filled glovebox oven.

Except some minor changes, the electrolyte remains stable under these heating conditions. In the next step, the pre-heating was repeated at 100°C with all other conditions unchanged. The TICs are presented in fig. S8.

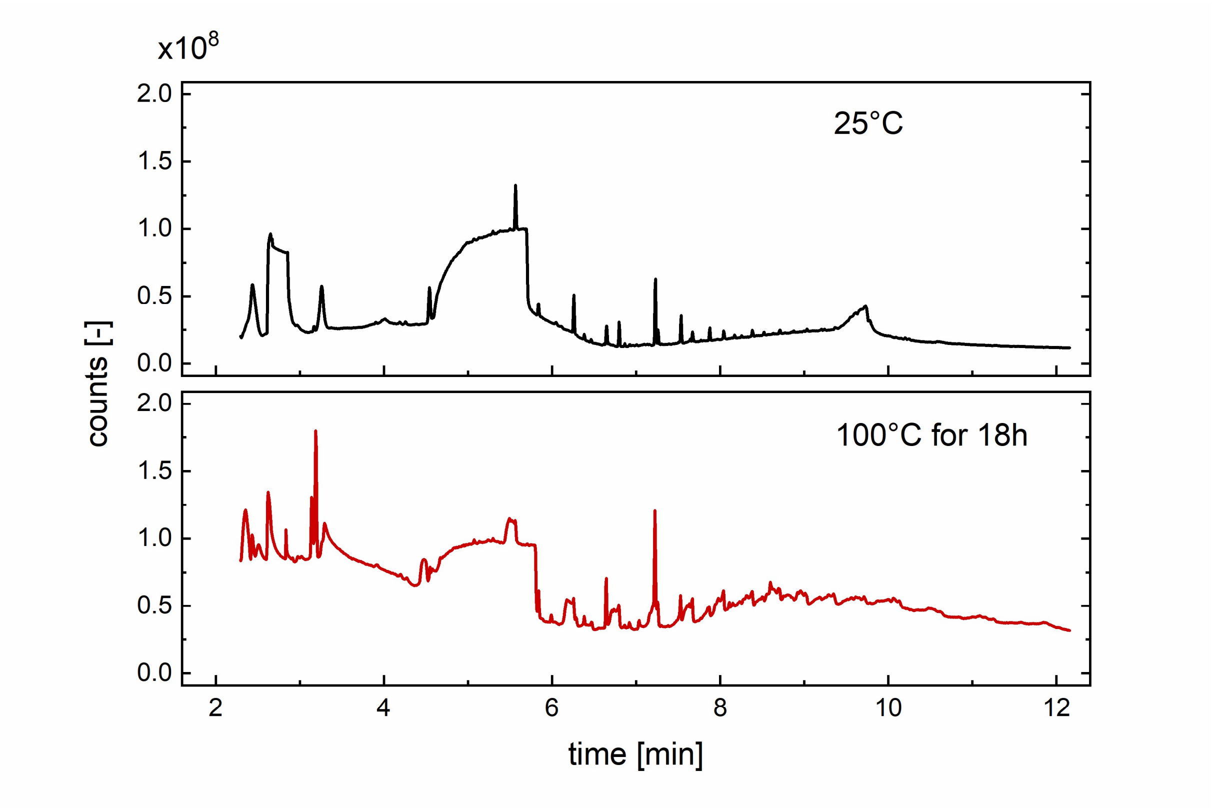

Figure S8. TICs of LP57 electrolyte without and with pre-heating at 100°C for 18h in an Ar-filled glovebox oven.

At the elevated temperature, severe decomposition of the electrolyte is notable. Due to its increased thermal stability, the broad EC solvent peak (4.5 to 6 min) is not significantly affected, whereas the EMC solvent peak (2.7 to 3 min) almost completely vanishes in a high intensity signal shoulder. Please note that differences between the pristine LP57 chromatograms arise from different batches used. The 100°C electrolyte sample also changed its colour from transparent to red (fig. S9).

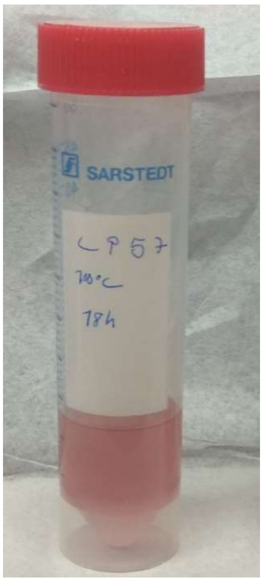

Figure S9. Colour change of the LP57 electrolyte after heating of 18h at 100°C.

Calibration of the reference electrode

Prior to each measurement the half-wave potential of the home-made reference electrode[5] (RE) was determined. The RE was put in a beaker cell filled with the electrolyte of interest (i.e. 1 M LiPF6 or LiTFSI in EC/EMC 3:7) and additional 3.3 mM ferrocene. Three electrochemical impedance spectra (10 Hz to 200 kHz with 10 mV sine wave amplitude) were measured to determine the solution resistance R_u with a 1 mm diameter glassy carbon rod as working electrode (WE) and a 250 µm diameter platinum wire as counter electrode (CE). Typical R_u values ranged from 10 to 50 Ohm (see figure S10 left). With the as-defined solution resistance four CVs were recorded from 2.1 to 3.7 V vs RE at a scan rate of 100 mVs^{-1} (see figure S10 right). The potential values of the anodic and cathodic peaks E_{pa} and E_{pc} were subsequently used to determine the half-wave potential $E_{1/2}$ of the electrode against the ferrocene / ferrocenium couple according to equation S10:

$$E_{1/2} = 0.5\ (E_{pa} + E_{pc}) \qquad \text{S10}$$

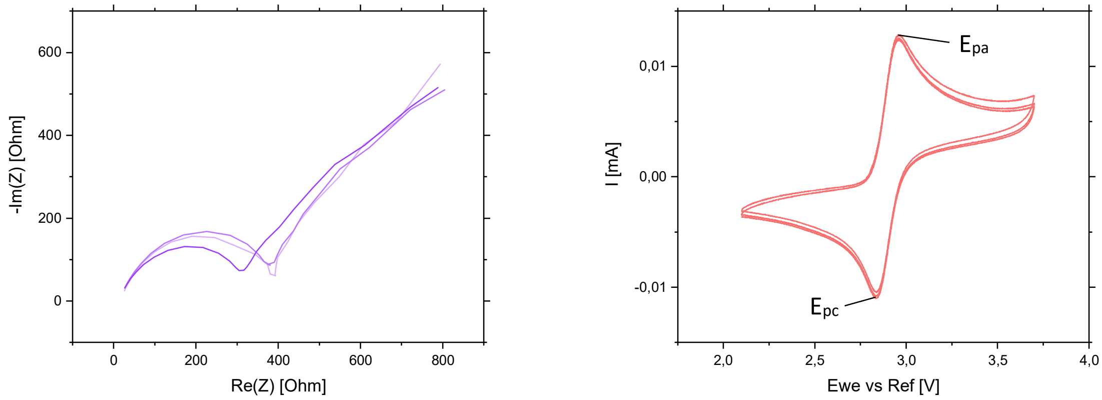

Figure S10. (left) Nyquist plot of impedance spectroscopy performed in an electrolyte / ferrocene mixture with glassy carbon as WE, platinum wire as CE and the home-made RE. The left-hand starting point of the graphs indicates the solution resistance R_u. (right) Cyclic voltammograms of the same beaker cell and IR-correction at 85% of R_u. The oxidation and reduction peaks Epa and Epc are marked.

From the works of Laiore et al.[6] and confirmed with our own setup it is known that the half-wave potential of a lithium reference electrode in the ferrocene / $LiPF_6$ electrolyte is 3.25 V; hence the half-wave potential of the home-made RE vs Li^+/Li may be calculated according to equation S11:

$$E_{1/2}\ (\text{V vs Li}^+/\text{Li}) = 3.25\ \text{V} - E_{1/2}\ (\text{V vs RE}) \qquad \text{S11}$$

References

1) Gnielinski, V. G4 Längsumströmte ebene Wände in: *VDI-Wärmeatlas, Springer Berlin Heidelberg,* **2013,** 805-808
2) Logan, E. R.; Tonita, E. M.; Gering, K. L.; Li, J.; Ma, X.; Beaulieu, L. Y.; Dahn, J. R. A Study of the Physical Properties of Li-Ion Battery Electrolytes Containing Esters *J. Electrochem. Soc.* **2018***, 165*, A21-A30
3) S.C. Chen; C.C. Wan; Y.Y. Wang Thermal analysis of lithium-ion batteries *J. Power Sources* **2005** *140(1), 111–124*

4) Wachs, S. J.; Behling, C.; Ranninger, J.; Möller, J.; Mayrhofer, K. J. J.; Berkes, B. B. Online Monitoring of Transition-Metal Dissolution from a High-Ni-Content Cathode Material *ACS Appl. Mater. Interfaces* **2021***, 13*, 33075-33082
5) Behling, C.; Mayrhofer, K.; Berkes, B. Formation of lithiated gold and its use for the preparation of reference electrodes — an EQCM study *J. Solid State Electrochem.* **2021**, 1-11
6) Laoire, C. O.; Plichta, E.; Hendrickson, M.; Mukerjee, S.; Abraham, K. Electrochemical studies of ferrocene in a lithium ion conducting organic carbonate electrolyte *Electrochim. Acta* **2009**, 54, 6560-6564
